# Assessing community health worker service delivery in humanitarian settings

**DOI:** 10.7189/jogh.10.010307

**Published:** 2020-06

**Authors:** Nathan P Miller, Adam K Richards, Melissa A Marx, Francesco Checchi, Naoko Kozuki

**Affiliations:** 1 UNICEF, New York, New York, USA; 2 Mailman School of Public Health, Columbia University, New York, New York, USA; 3 Division of General Internal Medicine and Health Services Research, University of California Los Angeles, Los Angeles, California, USA; 4 Bloomberg School of Public Health, Johns Hopkins University, Baltimore, Maryland, USA; 5 Faculty of Epidemiology and Population Health, London School of Hygiene and Tropical Medicine, London, UK; 6 Research, Evaluation, and Learning Unit, International Rescue Committee, Washington, D.C., USA

**Figure:**
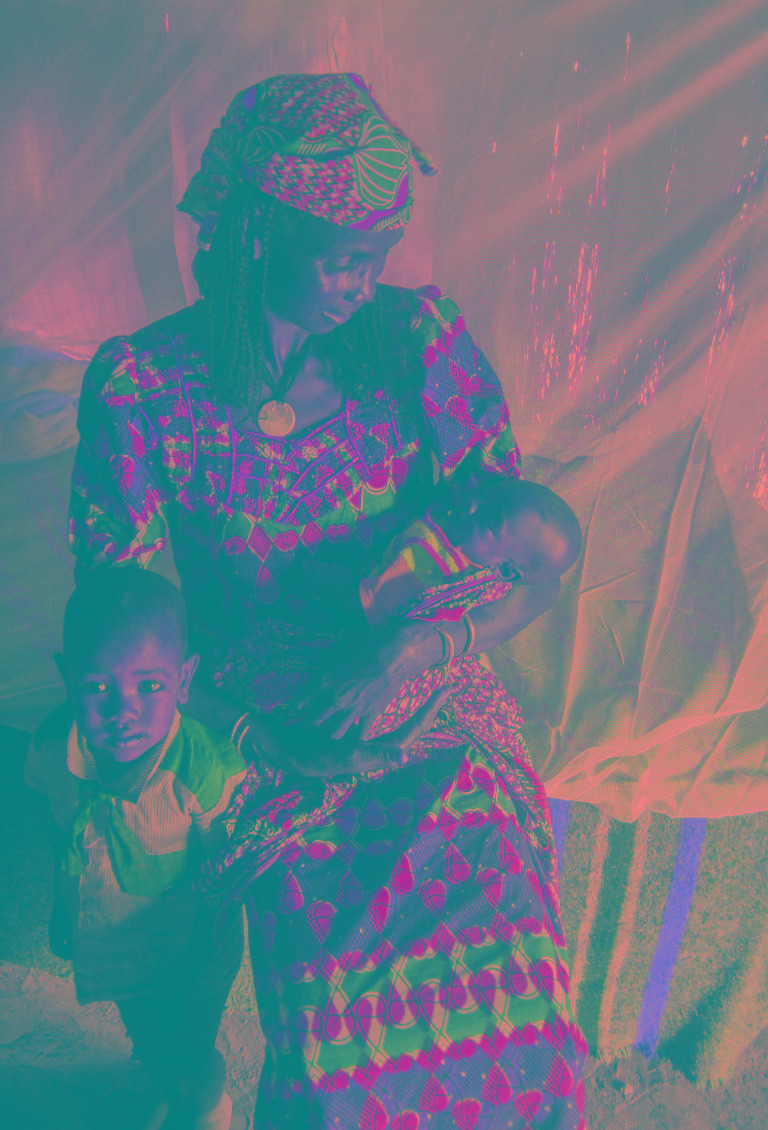
Photo: Refugees from the Central African Republic in southern Chad (from the collection of Nathan P Miller, used with permission).

In humanitarian emergencies, an urgent need arises to improve access to essential health care services, especially for affected populations who reside outside of camp settings and are thus less accessible through fixed health facilities. In conflict-affected contexts, with high levels of insecurity, population movement, disruptions in supply chains, and exacerbations of pre-existing shortages of human and financial resources, it is especially difficult to deliver services to affected populations. Building on the track record of community health workers (CHWs) delivering a range of life-saving health care services to hard-to-reach populations, there is renewed interest in supporting CHWs to deliver these services during humanitarian crises [1]. Existing evidence suggests that CHWs can continue providing routine services, as well as new emergency response activities, during crises [2-5]. There is now a need for rigorous assessments of the feasibility and effectiveness of delivering essential health services in humanitarian settings through community health systems.

To understand the degree to which community health services can be successfully delivered in an emergency, it is necessary to assess core indicators of implementation strength (eg, training, supervision, drug supplies, etc.), quality of care (eg, adherence to clinical guidelines), and utilization of services. In addition to measuring these quantitative indicators, qualitative data collection will help in understanding important barriers and facilitators to effective delivery of services. To demonstrate impact of services, it would also be important to measure coverage of interventions and health outcomes at the population level. Ideally, assessments of service delivery in humanitarian emergencies would adhere to the most rigorous methods used in non-emergency settings; it may not be necessary to develop different methods for a humanitarian context. However, the challenges of some emergency contexts do require adapted or innovative methods.

To inform the design of an evaluation of CHW service delivery in acute emergencies, we conducted a rapid review of the literature related to methods for assessing health service delivery in humanitarian settings. A number of key lessons were identified that can inform the future development of methods for assessing CHW service delivery in humanitarian settings.

## DEFINING HEALTH SERVICES AND STANDARDS

Assessments of health services in humanitarian settings often use inconsistent definitions of different health services and measure inconsistent indicators, making it difficult to compare and aggregate results. Standardized definitions exist for some health services, such as for malaria control and reproductive health, but more work is needed to develop standard definitions for the spectrum of essential health services [6].

## MAPPING OF HEALTH SERVICES

Checchi et al. highlight a need for health services and resources in fragile settings to be mapped out prior to a crisis. A database of health facilities, human resources (including CHWs), and services available should be developed. Further, identification of pre-crisis health system weaknesses and inequities will aid in quickly identifying priority areas for intervention when a crisis occurs [7].

## IMPORTANCE OF QUALITATIVE DATA

Health service assessments in humanitarian settings typically focus on key quantitative indicators. However, qualitative data provide crucial insight into service delivery challenges and identify areas for improvement. Qualitative data were reported to be particularly useful for obtaining perspectives of frontline health workers and beneficiaries and providing information to help interpret quantitative findings [8].

## USE OF MOBILE PHONES FOR DATA COLLECTION

A recent assessment of the strength and quality of Mali's integrated community case management of childhood illness (iCCM) program explored the feasibility, reliability, and validity of phone-based data collection. The study included one district where flooding and conflict severely restricted access to field sites. In this district, less than half (41%; 7/17) of CHWs were reached for in-person interviews, but all of the CHWs completed interviews by phone, equal to the completion rate in unrestricted districts. Validity and reliability were adequate for several indicators across the study sites, including in the restricted district. For these indicators, cell phone-based data collection could enable evaluation in otherwise inaccessible settings, including in emergencies [9].

## USE OF LOCAL DATA COLLECTORS

A study in Afghanistan illustrated the benefits of task-shifting data collection to locally-based data collectors for the balanced scorecard program. The use of local data collectors was a response to difficulties faced by the traditional survey teams, which were composed of native Afghans from other regions, to safely access health facilities in particularly insecure areas. The lack of security led to the exclusion of randomly selected health facilities in a large majority of provinces, leading to selection bias and a likely overestimation of performance. In response to these challenges, local community members were recruited and trained as survey data collectors to collect data in their home areas. Teachers were often selected to ensure a high level of literacy and availability in all areas of the country. These locally-based data collection teams were viewed as less intrusive and were able to assess facilities that the standard survey teams could not. Furthermore, the cost of using the local data collection teams was substantially less than the standard survey teams [10]. Local approaches to data collection have also been successfully used to measure health status and the impact of CHW interventions in “black zones” of other chronic conflict settings, such as Myanmar [11].

## TOOLS FOR ASSESSING COMMUNITY-BASED HEALTH SERVICES IN HUMANITARIAN SETTINGS

No data collection tools were found that were specifically for assessing community health service delivery in humanitarian settings. The World Health Organization has proposed the use of the Health Resources Availability Mapping System (HeRAMS) tool, which does include indicators for community health services. However, these indicators relate to availability of community health services and resources (eg, number of CHWs attached to health facility, population covered, and which services are provided). Most key implementation strength indicators at community level are not included in HeRAMS [6].

## CONCLUSIONS

There is a gap in evidence on how to assess community-based health service delivery in humanitarian settings. Methods, tools, and indicators need to be developed, tested, and made available for use in crisis-affected and fragile countries. In particular, there is a need for studies that test methods for assessing CHW implementation strength, service utilization, and quality of care in humanitarian settings where access to CHWs and populations may be limited. Further studies evaluating the use of rapid surveys, mobile data collection, or locally-based data collectors to collect these data would be welcome.

The methods used for assessing community-based service delivery in humanitarian settings may need to be flexible and to take into account the local context, which can change by the day in an emergency. For example, up-to-date and local information will be needed to determine whether data collectors can safely visit communities or whether CHWs can make it to a health facility for assessment. Given the security risks that CHWs, supervisors, and data collectors are exposed to in humanitarian settings, there is a need for improved methods for assessing security risks and evaluation of measures to reduce risks.

We encourage future investment in systematic documentation of experiences and methodological development by governments, implementing agencies, and academics to improve learning for humanitarian service delivery. This is also a call for harmonization of efforts. Standardized tools and common indicators of CHW implementation strength, quality of care, and utilization of services should be developed, tested, and disseminated to governments and humanitarian partners.
